# Neither ‘foolish’ nor ‘finished’: identity control among older adults with HIV in rural Malawi

**DOI:** 10.1111/1467-9566.12531

**Published:** 2016-12-24

**Authors:** Emily Freeman

**Affiliations:** ^1^ Department of Social Policy London School of Economics and Political Science London UK

**Keywords:** ageing, coping/coping strategies, developing countries, experience of illness, HIV, identity

## Abstract

Prevalence of HIV after age 50 is considerable, especially in southern Africa. Negative social constructions of HIV in older age, and the health consequences of ageing with the virus, mean that having HIV presents a challenge for many people's roles and social memberships, threatening to disrupt their sense of self. Using constructivist grounded theory and qualitative data from rural Malawi, this paper describes how older men and women deal with these identity challenges. Drawing on a symbolic interactionist framework, it uses identity control theory to explore how the study's participants presented their post‐diagnosis behaviours in ways that maintained their most significant pre‐diagnosis identities as ‘adults’, a label they gave to the core identity of being a person who belongs in the social world. Considering the processes through which older people with HIV navigated challenges to their identities in light of the intersectional influences of HIV and age‐related stigma and illness, provides insight into how older people might experience HIV, as well as informing theoretical understandings of identity formation and maintenance in light of chronic and/or stigmatising illness more broadly.

## Introduction

For more than 30 years, Human Immunodeficiency Virus (HIV) has implied a social identity, rather than just an illness (Sontag 1988). Even in countries with generalised epidemics, it is associated with sex and risk, and certainly in the not‐too‐distant past, the tragic deaths of people ‘taken too soon’. Across the globe HIV has typically been considered as a young person's disease. This is not simply a reflection of epidemiology or availability of data: it has been recognised for a decade that a considerable proportion of those with HIV are aged 50 and older (UNAIDS 2006). In the dominant global imagination, the risky sexual behaviours and outcomes associated with HIV are not those associated with older age. Older adults with HIV must therefore navigate the intersectional influences of HIV and age‐related stigma (Emlet 2006; Freeman 2016; Kuteesa *et al*. 2012).

In Malawi, HIV at older ages (50+) is high and increasing. It is likely to account for 18.6 per cent of the total adult HIV epidemic (Negin & Cumming 2010) and might be much higher among men. In Malawi's majority rural population, HIV among men aged 50–64 in 2010 was significantly higher than among those aged 15–49 (8.9% compared with 4.1%; Freeman and Anglewicz 2012). This HIV prevalence is attributable to both new infection in later life and increased survival of those infected earlier in life following expansion in access to effective antiretroviral treatment (ART).

Even with ART however, HIV remains a disruptive health experience. People aged 50 and older with HIV are likely to be at greater risk of ill‐health than younger adults with HIV, associated with declining functionality of the immune system, HIV‐related illness, non‐AIDS‐related illnesses and non‐communicable disease (Nguyen and Holodniy 2008). Moreover, in settings with compromised health systems like Malawi, ART remains limited: In 2012 30 per cent of Malawians who needed ART did not receive it (WHO 2013) and access to second‐line treatment in the event of treatment failure was extremely limited (Harries *et al*. 2010). Malawians with HIV of all ages therefore face the continued possibility of sickness.

The negative social construction of HIV and the health consequences of the virus mean that HIV changes older adults’ lives. It changes how they manage their daily lives, their relationships and their plans for the future. It changes what they can and cannot do. It changes how they are perceived by others and their social position. In short, it alters the interactions through which individuals make meaning about the world around them. Consequently, HIV may not only change older adults’ roles and social memberships, it may disrupt the social processes through which the self emerges (Blumer 1969).

The self and identity have been largely overlooked as an analytical category through which to explore how older adults make sense of living with an illness that is particularly stigmatising at older age. Nevertheless, experiencing HIV – both with and without the presence of ART – is widely understood to alter, question or undermine individuals’ identities; ability to navigate identity challenges is recognised as one of the key elements of psychological functioning required for managing life with HIV (Swendeman *et al*. 2009). Considering the processes through which older adults do this therefore provides valuable insight into the way they experience and manage infection.

Previous work on chronic illness and identity has typically viewed illness as an identity ‘disruption’ (Bury 1982) – a bodily failure that profoundly disturbs daily activities and relationships and subsequently an individual's realities, and/or as an identity ‘spoiler’ (Goffman 1963) – a damaging social identity that leads to a spoiled self‐identity, as once labelled, individuals internalise stigmatising discourses communicated through interactions. An individual with chronic illness is therefore expected to carry out what Corbin and Strauss (1987) have called ‘biographical work’ in the face of others’ altered perceptions of their behaviours and social identities. This work involves the ‘review, maintenance, repair, and alteration’ of an individual's biography in light of their changed reality (Corbin and Strauss 1987: 264). Consideration of the effect of HIV on the self has typically focused on younger adults’ experiences. In this regard, identity work is expected to include identifying and accepting which aspects of the self have been lost through experience of diagnosis and illness, which can be reclaimed and which aspects have been added, so that the self is recreated, or at least refined, in such a way as to ‘incorporate’ HIV (Baumgartner 2007, Russell and Seeley 2010, Seeley *et al*. 2012, Tewksbury and McGaughey 1998).

Both explicitly and implicitly, this scholarship is routed in symbolic interactionism. It centres on the processes autonomous actors use to create and re‐create their realities and definitions of self through interactions. Within these interactions, actors are continually interpreting the symbolic meaning of their environment (including the actions and expected beliefs of others) and act on the basis of this imputed meaning (Carter and Fuller 2015). For example, in rural Uganda, Russell and Seeley (2010) identify the incorporation of HIV into identity among participants of an ART programme through adaptive strategies of work and resource mobilisation. These behaviours and interactions enabled participants to practically rebuild their economic livelihoods following initial serious illness, while also regain a sense of control and ‘normality’.

The symbolic interactionist framework also underpins work to understand how participation in HIV activism has enabled individuals to construct new identities following diagnosis. Echoing interactionist scholarship on collective behaviour and social movements, Levy and Storeng (2007), Robins (2005) and Tsarenko and Polonsky (2010) trace how involvement in HIV support groups, ART campaign work and awareness raising activities, can facilitate interactions that promote social integration and group membership following collapse of social networks as well as empowering social work following status loss.

In this paper I describe how men and women navigate and respond to identity problems presented by having HIV in older age in rural Malawi. I discuss the nature of the age and HIV‐related stigma underpinning these identity challenges in full elsewhere (Freeman 2016). Continuing the tradition of applying an interactionist framework to understand the self, I draw on identity control theory (Burke 2007) to unpack the processes by which participants (re)constructed positive identities and avoided ‘spoiled’ identities.

## Methodology

Since social behaviours take place on the basis of subjective meanings (Blumer 1969), the study aimed to explore older adults’ experiences of growing old and of HIV infection from their perspectives, recognising the limitations of any interpretation of their experiences. It used constructivist grounded theory (Charmaz 2006) to generate and analyse data that privileged what older adults themselves presented as the most salient elements of their experiences. This design allowed for discovery in this under‐researched area.

With the help of local research assistants, I interviewed 43 older adults (20 men and 23 women) at their homes in rural villages in southern Malawi, most several times (N = 135). Interviews lasted one or two hours, although some were longer. Almost half of the participants (N = 18) had HIV, allowing me to explore how shared meanings of HIV in older age were produced. I also conducted three focus group interviews with older and younger members (aged between around 30–75) of three HIV support groups, each consisting of 10–15 participants. Reflecting the groups’ membership, participants’ ages were skewed towards old ages. Interviews were all conducted in Chiyao or Chichewa.

No specific hypotheses were identified at the outset of the research and the content of interviews varied between participants and over time (9 months between 2009 and 2010). Participants discussed relationships, illness, sex, politics, farming, death, love, poverty, growing old and HIV, among other topics. Emerging analytical ideas were discussed with participants to ensure their credibility. In keeping with grounded theory, I interviewed people and analysed their responses during the same period. Interview questions became more specific as my analytical ideas were explicated and relationships between them were examined. The importance of identity emerged from analysis of data generated; existing scholarship on identity was explored later, following the period of data collection and initial analysis. Similar to Charmaz's approach (1987) I sought to preserve the meanings of participants’ narratives while framing them in a sociological analysis.

Participants for individual interview were randomly recruited from stratified samples of older adults who had participated in the Malawi Longitudinal Study of Families and Health (MLSFH), which includes HIV testing (see Anglewicz *et al*. 2009) (n = 23), and purposively recruited from the families of existing participants (e.g. parents, spouses) (n = 9), HIV support groups (n = 9) and the local area (healers) (n = 2). I used theoretical sampling to identify these participants, who were aged between approximately 50 and 90 years. Sampling aimed to maximise variation in the analytical categories being developed so that experiences I expected might be different (e.g. because of age, health, living arrangements) could be juxtaposed and examined (Corbin and Strauss 2008). Samples were drawn based on HIV status, age and gender, as well as various relationships (e.g. marriage, living with older parents) and occupation (being a healer).

The study was described as being about growing older and HIV infection (generally) to potential participants, almost all of whom (minus three) consented to take part. Research assistants helped to recruit participants who attended HIV support groups and were subsequently aware of their serostatus. However they were not aware of the results of the HIV testing carried out by the MLSFH and did not ask participants about their HIV status, leaving those with HIV to introduce this information during the interviews if they wanted to. All participants known to me through MLSFH testing to have HIV did discuss their infection during our conversations. Three participants were taken to HIV counselling and testing centres at their request.

Participants were given small gifts when visited for a research conversation. These were presented to participants on greeting, irrespective of whether an interview was conducted and did not appear to influence participation.

Quotations in the paper, all from participants with HIV, appear alongside pseudonyms and approximate ages. Few participants knew their exact chronological ages. The majority of older adults with HIV included in the study appeared to be aged between 60 and 70 years old.

Permission to conduct research was granted locally by village heads, the regional branch of the National Association of People Living with HIV/AIDS in Malawi and HIV support group leaders, nationally by the National Health Sciences Research Committee as part of the MLSFH, and internationally by the London School of Economics and Political Science Research Ethics Committee.

## Theoretical approach

Participants said contradictory things over the course of a single conversation and multiple interviews. For example, they reported struggling to work, but at other points in our conversation, listed with pride the tasks they had completed. The complexity of their narratives makes sense however when they are viewed as identity performances, akin to those made during interactions outside of interviews (Goffman 1958). Participants used our conversations to flag their disadvantage while presenting favourably ‘who they are’.

Identity theorists understand the self as composed of multiple identities based on sets of shared meanings, sustained through interactions and held by individuals (Burke 2004). This sense of self may be partly based on being a member of social groups (e.g. women; a religious community) and the behaviours associated with particular memberships, as enacted in particular situations, and partly based on occupying particular roles (e.g. parent; researcher) and the behaviours associated with those roles, in particular situations (Deaux and Burke 2010, Deaux and Martin 2003, Stets and Burke 2000, Thoits and Virshup 1997). The shared meanings of these identities serve as a reference point for assessing the self against and form a so‐called ‘identity standard’ (Burke 1991).

Burke's (2007) identity control theory (ICT) is particularly relevant to research on psychological responses to HIV because it addresses the internal processes guiding individual thought and behaviour that operate when a person's identity is threatened. Both group and role‐based identities are considered within ICT. Individuals have numerous identities, and rely on them at different times. When a particular identity is relevant in a situation (becomes ‘activated’), the individual perceives the meanings implied by their behaviour, either through their own observations or through receipt of others’ appraisals of their behaviour. Individuals then compare these perceptions about who they are in the situation (the ‘situational inputs’), with the meanings held for a particular identity (the ‘identity standard’). Individuals verify their identities by controlling perceptions of their behaviours to ensure that they match the identity standard. That is to say, they will maintain the alignment by continuing to behave in the ways eliciting those perceptions of the self. This is the goal of the identity process.

If there is an interruption in the congruence between the meanings of the identity standard and an individual's behaviour – that is, a person comes to believe that they are not behaving like a particular identity (e.g. a parent) – an individual is expected to alter their behaviour (behavioural responses) or the perceptions of their behaviour (cognitive responses) to modify the situational input so that it is aligned with the identity standard. If the individual is unable to modify their behaviour or perceptions of it and the discrepancy persists, the discrepancy will act to slowly change the meanings of the identity standard itself (e.g. what it means to be a parent), in order to become more in‐line with perceived behaviour (Burke 2007).

The most obvious way a discrepancy between situational input and identity standard may arise is through a disturbance to the meanings in the situational input – a change in the perceptions of an individual's behaviour. As discussed, HIV/AIDS frequently brings about changes in behaviour and perceptions of the meanings of that behaviour, or changes in perceptions of the meanings of past behaviours. For example, disability associated with HIV‐related illness – or social isolation as a result of HIV‐related stigma – may restrict individuals’ ability to behave in ways perceived as matching their identities; sexual behaviours that before diagnosis were imbedded with positive meanings of virulence and vitality can take on new meanings of risk and foolishness after diagnosis.

An important element in ICT is that incongruence between the meanings of an individual's behaviour and the meanings of an identity standard evoke an emotional response such that ‘we feel distress when the discrepancy is large or increasing; we feel good when the discrepancy is small or decreasing’ (Burke 2006: 83). Identity interruption may be particularly stressful when the disrupted identity is more highly significant to the individual (Stets and Tsushima 2001). ICT therefore provides a unifying analytical framework to understand emotional responses to HIV associated with changes in perceptions of a person, based on their behaviours, as well as the day‐to‐day strategies people employ to cope with social experiences of infection.

In the following section I briefly outline the nature of the identity challenge presented by HIV in older age (see Freeman 2016 for fuller exploration). I then turn to the crux of the paper: the strategies older adults with HIV employed to control perceptions of the meanings of their behaviours in order to align them with their preferred identities.

## Analysis

### HIV in older age as a challenge to the self

Participants’ narratives include reference to multiple identities (e.g. woman/man, parent/child, comedian, Muslim). However, it was the role and social identity of ‘adult’ that was activated most frequently during research conversations about HIV in older age. It was this identity participants consistently returned to when discussing their experiences and expectations. The adult identity was the most salient identity individuals presented, transcending all other identities.

For participants, to be an adult, was to behave in ways that were interpreted as being productive. The emphasis on productivity in the standard for this core identity is rooted in participants’ rural livelihoods. In the fieldsite, ability to support oneself and one's family through primarily body‐centred agricultural production, and ability to invest in relations of social interdependence that underpin access to farming land and familial and community support, is essential for the survival of individuals and households. Old and young carry out material and social production that includes farm work, paid work and housework (including caregiving, advising, sex and reproduction). Since life depends on productive activity, the very act of working is understood to demonstrate one's status as a living person and involvement in social life. To be an adult is to be ‘someone’.

During our interviews, participants commonly discussed the adult identity with reference to the counter‐identity of ‘child’. Both identities and participants’ labels for them are distinct from chronological or biological age. Throughout the interviews they were used by participants to emphasise the power(lessness) and social inclusion or exclusion of those normally considered ‘grown‐up’ (e.g. anyone aged 16 or older). Typically only the oldest old and the extremely sick were perceived to occupy the child‐like identity.

However, older men and women without and with HIV, presented HIV in older age as a challenge to attaining the adult status. Participants reported that regardless of age (i.e. whether 50 or 90), HIV undermined an older person's ability to contribute meaningfully to individual or household survival through material or social production. HIV accelerated older adults’ decline to the out‐group of the child‐like. Challenges were two‐fold, mirroring the most persistent understandings of HIV in later life.

First, in 2010 the perception that HIV is fatal remained prevalent. Despite the possibility for good health brought by increasingly available ART, almost everyone had witnessed AIDS deaths that were hard to forget. HIV‐infected older bodies, understood to be already weakened by age, were expected to be ‘finished’ – incapable of the physical work required for self‐sufficiency or familial contribution, and eventually even instrumental self‐care such as washing and feeding. The meanings of such incapacity did not correspond to those held in the adult identity standard. Further, as ultimately fatal, HIV infection in later life questioned an individual's very physical viability, the core requirement of social membership.

Second, the transmission of HIV through risky non‐marital sex in the face of pervasive HIV prevention messages, understood to be avoidable in older age, indicated to participants that older adults with HIV were ‘foolish’ and impulsive. HIV infection of older adults, typically the custodians of advice, was understood to follow from having not heeded others’ good advice. Further, since HIV in later life was understood to be acquired through non‐marital sex, those with HIV brought the virus into an otherwise infection‐free home. Meanings of these behaviours were incongruent with the meanings held within the adult identity standard: infected older people could hardly be considered socially productive, and having putting their families at risk of infection, were not acting with the familial providence and care expected.

### Resisting identity challenges

Although all participants recognised the challenge to adulthood presented by having HIV in older age, participants with HIV did not accept the non‐adult identity for themselves. They stressed that they were neither ‘finished’ nor ‘foolish’. The child‐like counter identity was instead available as a ‘possible self’ (Markus and Nurius 1986) – a negative identity other older people with HIV had, and that they too could have if they were not able to resist the challenges presented by HIV in older age. Markus and Nurius's theory of possible selves focuses on the dissonance between current identity and future identity, arguing that possible selves thereby become incentives that guide behaviours, thoughts and strategies. In this case, the unattractive connotations of the child‐like identity incentivised older adults with HIV to assert their adulthood.

Throughout the interviews, participants used narratives or stories as a key resource by which to resist the stigmatised child‐like possible identity that might be imposed on them. In‐line with identity control theory, two discursive strategies can be identified in participants’ narratives: participants either reported behaviours that were widely aligned with the adult identity standard, or managed perceptions of their behaviour in order to align them with the adult identity standard.

Although separated below for ease of presentation, participants’ narratives were nuanced and sometimes contradictory, reflecting the complexities of the identity work they were engaged in. Participants typically employed a combination of strategies across the course of our interviews, responding differently to the identity challenges presented by limitation of their material and social productivity (being ‘finished’ and ‘foolish’). The availability of (re)constructed positive adult identities was shaped by participants’ pre‐diagnoses identities, as well as the viability of claiming continued material or social production in light of their behaviours.

### Responding to perceptions of being ‘finished’

#### Stressing behaviour aligned with the adult identity

Despite frequent discussion of HIV's socially‐expected and sometimes‐experienced action on the body, no participants with HIV considered themselves to be ‘finished’. Their interview narratives can instead be viewed as performances of the adult identity. Their positions as viable and contributing members of the social world were demonstrated in discussion of behaviours and attitudes that emphasised realised or potential productivity.

The physically demanding work participants carried out on their farms and in their homes seemed a particularly important part of the stories they wanted to tell. They presented this work in terms of continuity with their pre‐infection behaviours. While a few participants, particularly those recently diagnosed, were quick to dismiss any suggestion of change in their ability to be productive (‘I don't have any problem … I just feel normal life as before’), for many initiation of ART had been a significant turning point. In the following example, Zione discusses how her life has improved now that she is able to work following treatment:

Last year I had problem, I was not good. I was reduced to a beggar. This year I have problems but they are not that much … if you [grow] your own food … You also have *people looking at you as a someone* … [when] I can do something to support myself, *I feel I am someone*. (Female, late 70s/early 80s, my emphasis)

Older members of HIV support groups, interviewed together, stressed their ‘commitment’ to hard work as a direct response to the discourse of finished HIV‐infected older people they perceived:

In the past we used to think when a person is sickly, we used to say they have HIV. The HIV people were not working, they were only waiting to be assisted … But here we are encouraging each other to be committed to doing work and depending on ourselves.

Throughout participants’ narratives, their material productivity is presented as demonstrating their self‐sufficiency and viability. The meanings of their behaviour are aligned with those held within the identity standard for an adult: Zione repeats that now she can support herself, she is a ‘someone’.

#### Managing perceptions of behaviour to align them with the adult identity

Nevertheless, some older adults with HIV were not able to claim the productivity widely understood to be aligned with the adult identity. Even following initiation of ART, they endured periods of short‐lived illness or prolonged disability. These participants could not work as they had previously.

Across our conversations these participants stressed that their limited material productivity was not really ‘them’: their behaviour did not reflect their identities. A dominant narrative for these participants was the distinction that could be drawn between their past, present and future behaviours. When asked why they were unable to work as they once had, participants reported that they were limited by illness associated with HIV rather than very old age, understood to imply an advanced unproductivity:

I was strong in the past … but my body is weakening … I am an old person, but not very old, it is only the disease which has weakened me … If I did not have the virus I would have been working a little bit. (Fiskani, male, 61)

By attributing declines in productivity to HIV, participants were able to distance themselves from being ‘finished’ since (in these discussions at least) participants understood that HIV could be effectively managed with ART, providing the possibility of future productivity. ‘Very old’ age in contrast, could not be reversed and inevitably led to a child‐like status. In the following example, Esnart make sense of her present behaviour (‘not doing anything’) in light of her previous behaviour (‘I was able to work’) by drawing on her identity as an independent adult and the possibility of behaviour that would reaffirm that identity in the future (‘one day I will be strong’):

In the past I was strong. I was able to work. I was pounding, fetching firewood in the mountain, drawing water. Before I was sick, I was very strong. But since 2003, I was not doing anything. Better this year, I can do a little bit … Even though I am weak now, I know one day I will be strong as I was in the past. (Female, mid‐60s)

The narrative of bodily decline through (managable) illness rather than very old age and the subsequent continuity of the ‘person inside’ was used strikingly by Daniel. Aged in his early 50s, Daniel lived on the edge of a tarmac road, a short distance from the closest rural trading centre. This was some miles from other participants whose homes were much less accessible. He was one of a few participants to have a business outside of agricultural production. He owned a tea room: a small building with a tin roof containing a wooden table and bench. There were no customers during any of our visits.

Asked to describe himself at the beginning of our first interview with him, he answers:

OK, at the moment I am married. My main occupation is farming, but I love to do business. At the moment my business has gone down, because of the disease. I started becoming sick in 2003; [200]4‐5, is when I started receiving the ARVs. The child that I have in this marriage is only one son, but I found my wife with four children, I found them very young, it's me who has brought them up.

In the passage, Daniel implies continuity in his behaviour by stressing that he is only unable to work ‘at the moment’ and quickly highlights his past physical (farming and business) and social (bringing up children that are not his) productivity. In immediately focusing on the temporality of his behaviour and the context of his past behaviour, he persuades us to recast his current behaviour and likely future behaviour as compatible with adulthood.

We next saw Daniel a couple of weeks later. Asked how he had been, his answer can again be understood as an attempt to manage perceptions of his HIV‐related behaviour so as to align it with his preferred identity:

This week the most time was spent on my illness. But I really wanted to be farming. So that my wife should be left here [at the tearoom] and I start farming little by little. So this week … I spent much of my time in bed. I have spent all the two weeks in bed. Previously I was trying to do some other things.

He presents this period of illness within a framework of productivity and effort. The time he spent in bed is time he would have liked to have spent working (‘I really wanted to be farming’) and time he would previously have spent working (‘trying to do some other things’). In doing so, I suggest he highlights to the interviewer what kind of person he is: being in bed is not typical behaviour for him. As in the previous interview, he suggests the continuity of his productive behaviour over time: he was working previously and through emphasising ‘this week’, implies that inability to work is not permanent and he may return to work the following week.

Later in the interview he emphasises that he is a business man. He stresses the size of the plot for his tea room and that due to his business acumen the tea room is still functioning. Although he lacks capital investment, like Esnart, he has the potential to be productive: he is in ‘a good business place’:

This place belongs to somebody [else] but all this part here is ours. Yes I have a big place even behind here … All this yard from the fence to that end is my place … The problem is the capital … The business is there, if one can have a good capital then you can be able to gain a lot … So there are a lot of problems but we are at a good business place.

### Responding to perceptions of being ‘foolish’

If not finished, participants with HIV certainly did not perceive themselves as foolish. They frequently established this using (unsolicited) narratives about how they contracted HIV. Just as participants without HIV, participants with HIV distanced themselves from the younger adults and unwise older people who infected themselves and their families through their unrestrained sexual behaviours. In othering those who were not sexually restrained and documenting their own behaviours of wisdom, care and providence, participants reaffirmed their social identities as members of the in‐group of adults. Participants reported either continuity or change in their behaviours in ways that aligned them with the shared meanings and expectations associated with the adult identity and its performance.

#### Stressing behaviour aligned with the adult identity

Some participants took pains to explain how they had contracted HIV through non‐sexual routes: caring for someone sick with HIV (informally within the household, or formally as a *sing'anga* [healer]), delivering the baby of someone with HIV or preparing the body of someone who had died of AIDS for burial. These non‐sexual behaviours are not foolish or irresponsible. They are widely understood to indicate wisdom and considerable knowledge, social responsibility, selflessness and compassion. In their narratives it is their social roles and behaviour as adults that put them at risk of HIV infection:

You know I prepare dead bodies. When they hear a cry, they say go and get [Stella] to come and help in preparing dead bodies. Mercy lost a person's life. (Stella, female, 70s)

Other participants identified sexual routes of their infection, but dated their sexual behaviour to before they knew of the risk of HIV transmission. Since it was not sex in general that was foolish, but sex when there were known health‐risks (see Freeman and Coast 2014), such sexual behaviour was therefore not associated with being unrestrained or irresponsible:

The disease I had, I did not know that it can be HIV … I did not know very well, about *kugonana* [sex outside marriage, lit. sleeping together], touching one's blood, and other means, I didn't know about them. (Fiskani, male, 61)

Participants describing either HIV transmission route stressed that their behaviour had not changed: they continued to act as caregivers and according to known sexual risks. However they constructed their interview performances to ensure observers recognised that the meanings of their behaviour were aligned with the meanings of social and material productivity held within the adult identity standard.

#### Managing perceptions of behaviour to align them with the adult identity

Other participants emphasised change in their behaviours in a way that encouraged us to perceive contrasting pre and post‐diagnosis behaviours as equally congruent with adulthood. Most commonly, participants’ narratives situated past ‘risky’ sexual behaviour within a discourse of sexual capacity aligned with the physical productivity expected of adults. Current sexual behaviour was alternatively presented as demonstrating wisdom, provision for family and self‐restraint, behaviours aligned with the social productivity expected of adults.

For example, in the following passage, Steven uses the change in his behaviour following HIV diagnosis to illustrate his shift from sexually capable (physically powerful) adult to still‐capable, but sexually‐restrained (socially powerful), adult. He emphases the authenticity of this identity by stressing that he and others recognise him as occupying this role (‘I am’; ‘my friends call me’):

I had been doing a lot in youthful life, including having many women – though I was not very promiscuous: I would only have one at a time, [although] I did have a lot of girlfriends … I was having sex with two or three women every 10 years … I still maintain my target: I am still going at every 3 years, I am having [sex with a different] woman. This time I am 57 years, but I have been with 17 women … I used to [have sex] with girls, but now I just see them as my friends, even my friends call me ‘friend of girls’. (Steven, male, 57)

Steven had lost his first wife and great love around five years before the interview. His stories of starting an extra‐marital relationship around the same time as remarrying provide another example of participants’ identity work of shifting perceptions of their behaviour. Believing himself to have become infected from one of his two new partners, he presents his failure to insist both women went for HIV testing before starting their relationships as a rashness borne of adult‐like desire to care for them, rather than child‐like sexual impulsiveness. Although Steven's affair is on‐going and remains a secret from his wife, he presents the relationship as a morally and socially justifiable love relationship entailing social and material providence. In doing so he is able to position both his past and present behaviour as fully congruent with adulthood. Despite initially claiming to have become infected by one of the two women rather than his first wife (whom he explained had had an affair he never got over), throughout our conversations he emphasises the steps that he had taken to protect the health of his partners (thereby avoiding introducing HIV into a household) and his plans to care for them:

I very much fell in‐love …When I am not home then it means I am with that woman at her home … I take her as my second wife … even if she will be found HIV positive I will never leave her, she will still be mine. (Steven, male, 57)

### Resisting the ‘finished’ and ‘foolish’ child‐like identity through living positively

Whether emphasising continuation or change of behaviours, all participants with HIV discussed managing their infection as ‘living positively’. Globally, positive living has offered a narrative account of life with HIV that has aimed to transform infection into an optimistic outlook and a way of managing the virus. Locally in the fieldsite, the discourse is embedded within HIV advice and counselling, and mutual‐support and advice circulated within HIV support groups. Participants’ accounts of positive living accord with those observed elsewhere: for them it involved ‘accepting’ one's HIV diagnosis to gain access to the treatment and care available, strengthening the body by eating a varied, protein‐rich and higher calorie diet, and preserving the body by limiting vigorous physical work (including sex and farming) and making sustained efforts to reduce stress.

However, older people in this study were not passive recipients of the global positive living narrative. Instead they produced and reproduced it in ways that allowed them to control their salient adult identities. For participants, living positively involved behaviours and attitudes that mirrored those recognised as materially and socially productive. Despite the apparent solidarity of support group communities, in private individual interviews, participants used the narrative to differentiate themselves from younger adults with HIV whose behaviours might more easily be seen to conform to dominant associations between HIV and being finished or foolish. They were, they reported, simply better at living positively.

In their interview narratives and stories, participants highlighted a set of behaviours that indicated they were more successful in managing HIV than younger adults. They were ‘not proud’, they recognised good advice, were resilient to others’ negative perceptions of them, were better able to frame HIV positively and were not interested in risky sex:

I can think, as for me, to be found with a virus like this one, which disturbs your body, it is better for an old person … Because we old people do take care of ourselves … These young women, they haven't finished [having sex], the young men they haven't finished [having sex] … Eee! Concerning *chiwerewere* [sex outside marriage] they are not finished with it. They are still sleeping together, but for us the [older people] … We just say my life should continue. So that I can do this job and that [e.g. farming and housework], you can't think of a man. (Esnart, female, mid‐60s)

The absence of these qualities and behaviours prevented younger adults from attending HIV support groups:

I: In your group, who are many, the old people or the younger people?

R: The youth do not come, maybe they are ashamed … They feel they will be looked down upon by people, they want people to respect them … but with us we just walk freely, without pride, we don't mind anything. (Esnart, female, mid‐60s)

Using these behaviours, participants demonstrated to observers (both us and those around them) their social and physical viability – behaviours congruent with the adult identity. Here Susan outlines her social production (wisdom, respect for advice, caregiving) and subsequent physical survival (status as a living being):

I am an old person. I was told I have the virus. I do follow what I was told at the hospital, so I stay and have sat down. But the young women do not want to believe it. They just say it has its time. So they continue to do what they were doing … For old people, they believe what they have been told. They say let me sit down, and be taking care of my children. That's it. […] I got the virus a long time ago but I am still alive because I listen to what I get from the hospital, what I get from the support group. (Susan, female, mid‐60s)

Further, in their narratives participants’ played a role in managing not just their own HIV infection, but also the HIV infection of others. They were actively involved in HIV prevention and care efforts, ‘sensitising’ others to the needs and experiences of people with HIV and encouraging testing, particularly among the very ill. Despite their grounding in HIV infection, otherwise considered to undermine participants’ social identities as wise and productive adults, these social roles shared the meanings associated with the adult identity.

In the following example, Nyuma, in a familiar story among infected participants, discusses how she has visited sick people and advised them to seek HIV testing and treatment. She ‘encourages’ these people, and receives thanks from their families. In doing so, Nyuma fulfils the roles of an adult: she contributes to individual and household survival. In this excerpt she outlines her care and advice‐giving. Like Steven (above), she stresses that her identity has been affirmed by others who recognise the value of her contributions:

R: She was sick, and she came here and slept here. When I asked her ‘what is the problem?’ she said ‘my mother is not caring for me very well’. I said ‘okay’. I took her to [name] Hospital. I said ‘Agnes, this is your patient’– that's my doctor … She was well received and helped. It's there where they told her, you have [HIV] … Then I took her to her mother, I told them, ‘here is your child, we are coming from the hospital’. They said ‘thank you very much, you have done a very good thing’ …

I: When you took this person to the hospital and caring for her, how did you feel?

R: I was feeling happy, I knew I was taking care of my friend. I had to show my love to her. I gave her water to bathe, and then took her to the hospital.

I: When you took her back to her parents, what//

R: [Interrupting] //They were very happy, happy indeed, they said ‘thank you, thank you very much! Our daughter when leaving here didn't tell us she was coming there, thank you very much, you have helped us a lot’, they said so! (Nyuma, female, 68)

Let us return to Daniel for a final example. His case highlights well the contrasts and complexities in participants’ identity work, as they shift attention away from discussion of their limitation in one kind of productivity to focus on their success in another kind. In the following excerpt, Daniel talks of his role as ‘mouth piece’ for the members of his HIV support group. In direct contrast to the meanings of powerlessness, non‐contribution and social exclusion associated with the ‘child‐like’ counter identity called to mind by his discussion of his health and inability to work, he presents himself as an integrated socially and materially‐productive member of a community, and a strong force to be reckoned with:

I really want those with the virus here to be helped. I am representing them all here at [place] … the radio has announced the allocation for those with the virus but we will not get that allocation which is in billions not millions, but it will end up in some other people's pockets in the offices and buying cars … Where is the help? They are not helping us. *Go and tell them that Daniel Mhango Mstogoleri has said so!* (Daniel's emphasis).

## Discussion and conclusion

In rural Malawi where supply of ART is limited, HIV remains associated with sex, illness and death. Consequently in Malawi, and elsewhere in Africa, an HIV diagnosis is widely understood to be a disruptive experience that influences not only an individual's day‐to‐day life, but also their identity. This paper has examined older people's responses to the challenges to their identities made by both the socially constructed institutions of old age and HIV, and the physical limitations of life with HIV as a chronic illness.

The narratives drawn from multiple interviews with 43 older people with and without HIV reveal a great deal about the identity work those with HIV have undertaken in order to manage their social status as HIV‐infected individuals and the implications of changes in their functional ability.

Although participants with and without HIV discussed HIV infection as undermining older people's positions as ‘adults’, an identity aligned with the status of being and belonging to the social world, no older people with HIV identified themselves as fulfilling the ‘child‐like’ counter identity. Instead adulthood was the most salient identity participants forwarded when discussing ageing and HIV, transcending all other identities. Across our conversations they used stories about themselves to align perceptions of their behaviour and the meanings held within the identity standard for an adult. Countering perceived accusations of being ‘finished’ or ‘foolish’, at different times and frequently linked to their explorations of feeling vulnerable, participants presented themselves as powerful, wise, caring and restrained actors capable of the material and social productivity expected of ‘adults’.

Although participants in this study reported new behaviours in response to their changed situations, the identities they forwarded as most salient to them were not new. By looking at the meanings and perceptions of behaviours, rather than just the behaviours themselves, considerable cogence between the meanings of the post‐diagnosis behaviours stressed by participants, and the meanings held in the identity standard for the pre‐diagnosis adult identity (as originally defined), can be identified. While participants had incorporated HIV into their lives – for example by engaging in HIV activism or by reducing their physical labour – this was not, for them, evidence of the acceptance of a new ‘HIV/AIDS identity’ (Baumgartner 2007, Levy and Storeng 2007, Robins 2005, Russell and Seeley 2010, Tewksbury and McGaughey 1998, Tsarenko and Polonsky 2010). In contrast to previous scholarship that has considered the impact of HIV and chronic illness on a wider range of identities, interviews with older adults in Malawi do not contain evidence of participants’ reflection on, or recognition of, the loss or gain of aspects of the self. Rather, they document narrative strategies in which participants played out cognitive and behavioural responses to identity threats in order to maintain the same self – their core, salient identities as ‘adults’.

The identities participants performed following infection influenced their experiences of HIV. By linking efforts motivated by avoiding negative possible selves to the day‐to‐day management of identity, we are able to explore how these older people with HIV were able to sustain positive identities over time, even when faced with episodes of ill health and negative social interactions resulting from their HIV infection.
